# Reply

**DOI:** 10.1002/hep4.2111

**Published:** 2022-10-10

**Authors:** Manuel Mendizabal, Nicolás Ducasa, Paula Benencio, Mirna Biglione, Ezequiel Mauro

**Affiliations:** ^1^ Hepatology and Liver Transplant Unit Hospital Universitario Austral Pilar Argentina; ^2^ Instituto de Investigaciones Biomédicas en Retrovirus y SIDA, CONICET‐Universidad de Buenos Aires Buenos Aires Argentina; ^3^ Liver Transplant Unit and Liver Unit Hospital Italiano de Buenos Aires Buenos Aires Argentina

To the editor:We appreciate the interest garnered by the article “Heterologous adenovirus‐vector/messenger RNA regimen is associated with improved severe acute respiratory syndrome coronavirus 2 humoral response in liver transplant recipients.” The comments by Dr. Sookaromdee and Dr. Wiwanitkit highlighted the importance of assessing prior severe acute respiratory syndrome coronavirus 2 (SARS‐CoV‐2) infection when testing antibody response to SARS‐CoV‐2 vaccination. This observation is relevant given that patients who recovered from corona virus disease 2019 (Covid‐19) present detectable prevaccination anti‐spike immunoglobulin G titers, even liver transplant recipients.^[^ ^1^ ^]^ Moreover, after receiving a complete vaccination scheme, the antibody titer can significantly increase compared to patients naive for Covid‐19.^[^ ^1^, ^2^ ^]^ However, to avoid this bias and consequently misinterpret the effect of vaccination, in our study population, we excluded patients with prior infection confirmed by polymerase chain reaction. Likewise, as detailed in the methods section, before patients' inclusion, antibodies toward the nucleocapsid (N) protein were analyzed by chemiluminescence assay (ALINITY SARS‐COV‐2 IGG; Abbott) in a certified biochemistry testing laboratory to estimate past SARS‐CoV‐2 infection.

In summary, we are satisfied with these discussions and interactions, which enrich our study, and we trust that our results will be a key tool for both conventional clinical practices as well as future research.

## CONFLICT OF INTEREST

Nothing to report.
